# Role of microRNAs involved in plant response to nitrogen and phosphorous limiting conditions

**DOI:** 10.3389/fpls.2015.00629

**Published:** 2015-08-13

**Authors:** Giao N. Nguyen, Steven J. Rothstein, German Spangenberg, Surya Kant

**Affiliations:** ^1^Biosciences Research, Department of Economic DevelopmentHorsham, VIC, Australia; ^2^Department of Molecular and Cellular Biology, College of Biological Science, University of GuelphGuelph, ON, Canada; ^3^Biosciences Research, Department of Economic Development, AgriBio, Centre for AgriBioscienceBundoora, VIC, Australia; ^4^School of Applied Systems Biology, La Trobe UniversityBundoora, VIC, Australia

**Keywords:** microRNA, fertilizer, nitrogen deficiency, phosphorous deficiency, nitrate and phosphate interaction

## Abstract

Plant microRNAs (miRNAs) are a class of small non-coding RNAs which target and regulate the expression of genes involved in several growth, development, and metabolism processes. Recent researches have shown involvement of miRNAs in the regulation of uptake and utilization of nitrogen (N) and phosphorus (P) and more importantly for plant adaptation to N and P limitation conditions by modifications in plant growth, phenology, and architecture and production of secondary metabolites. Developing strategies that allow for the higher efficiency of using both N and P fertilizers in crop production is important for economic and environmental benefits. Improved crop varieties with better adaptation to N and P limiting conditions could be a key approach to achieve this effectively. Furthermore, understanding on the interactions between N and P uptake and use and their regulation is important for the maintenance of nutrient homeostasis in plants. This review describes the possible functions of different miRNAs and their cross-talk relevant to the plant adaptive responses to N and P limiting conditions. In addition, a comprehensive understanding of these processes at molecular level and importance of biological adaptation for improved N and P use efficiency is discussed.

## Introduction

In the past half century, there has been a marked increase in food production allowing a significant decline in food shortages worldwide although the world population has doubled during this time (Godfray et al., 2010). However, in the next half century to achieve a similar expansion of food production to meet the needs of the increased human population is quite challenging given that with the confounding factors of narrowing arable land due to urbanization, a shortage of water for irrigation, global climate change, changing human diet, and significant increase in the proportion of food used for feeding animals and livestock or for producing biofuels (Rothstein, 2007). Cost effective approaches to increase crop production include but are not limited to the usage of modern high yielding crop varieties including genetically modified crops to increase productivity per unit of cultivated land, and the application of advanced agricultural practices such as minimal soil tillage and improvements in water and fertilizer use efficiency (Good et al., 2004). Improved fertilizer use efficiency requires balanced fertilization with adequate macro-nutrients and micro-nutrients (Baligar and Fageria, 2015).

Among macro-nutrients, both nitrogen (N) and phosphorus (P) are key elements for crop production and are major constraints for plant growth, development, and yield since intensive crop production relies heavily on a large input of these fertilizers (Vance et al., 2003; Sinha et al., 2015). Annually it requires approximately 85–90 million tons of N and 50 million tons of P fertilizer for crop production, worldwide (Good et al., 2004; López-Arredondo et al., 2014). However, crop plants are only able to exploit up to 40% of the applied N and P fertilizers, while the residual is lost to the environment through leaching, surface runoff, hypertrophication, denitrification, volatilization, and microbial consumption (Poirier and Bucher, 2002; Good and Beatty, 2011). Under the current trend, there will be about 2.5 fold increased level of eutrophication caused by excessive use of N and P fertilizer by 2050 (Poirier and Bucher, 2002). All these factors lead to a greater production cost and very significant levels of environmental pollution (Giles, 2005). However, it is pertinent to note that about 70% of arable land worldwide are deficient of Pi (Hinsinger, 2001; Kirkby and Johnston, 2008). Unlike N fertilizer which can easily be produced from the unlimited ambient N_2_, natural P resources such as phosphate rock, apatite used to manufacture P fertilizers are non-renewable and increasingly limited making it a major challenge for sustainable crop production in the future (López-Arredondo et al., 2014). It is estimated that an increase in nitrogen use efficiency by 1% worldwide, would save approximately $1.1 billion annually (Kant et al., 2011a). Therefore, it is of urgent importance to develop crop varieties with higher fertilizer use efficiency.

Plant small RNAs are short non-coding RNAs, which can be classified into two major groups based on their origin and biogenesis (Axtell, 2013). Small RNAs that are generated from perfect double-stranded RNA precursors are referred to as small interfering RNAs (siRNAs), which can be further divided into several subclasses such as heterochromatic siRNAs (hc-siRNA) and trans-acting siRNAs (ta-siRNAs) (Fei et al., 2013). Small RNAs that are processed from a partially double-stranded region of single-stranded RNA precursors are known as microRNAs (miRNAs) (Jones-Rhoades et al., 2006; Voinnet, 2009). Interestingly, miRNAs can trigger the production of secondary siRNAs such as ta-siRNAs (Voinnet, 2009; Fei et al., 2013). In this review, we focused on the involvement of miRNAs in the regulation of plant adaptation responses to nutrient deficiency. Readers who have interests on other classes of plant regulatory small RNAs, their biogenesis and modes of action are referred to other excellent reviews (Axtell, 2013; Fei et al., 2013; Patil et al., 2014; Weiberg et al., 2014; Kamthan et al., 2015) and the references cited therein.

MiRNAs have been identified as potent regulators of plant growth, development (Jung et al., 2009; Sun, 2012; Wu, 2013) and stress-responses including biotic and abiotic stresses (Phillips et al., 2007; Khraiwesh et al., 2012; Sunkar et al., 2012; Ferdous et al., 2015). In the last decade, miRNAs have also been implicated in nutrient uptake, transport and assimilation. Moreover, miRNAs were also identified as signaling molecules between cells, tissues, and organs (Chitwood and Timmermans, 2010; Meng et al., 2010). MiRNAs are short (19–24 nucleotides), single-stranded, non-coding RNAs and serve as post-transcriptional regulators of gene expression in plants (Jones-Rhoades et al., 2006). They are initially transcribed from MIR genes by RNA polymerase II to form primary-microRNAs (pri-miRNAs) with stem loop structures. A DICER-LIKE 1 protein (DCL1) processes these long pri-miRNAs at stem loop regions to form pre-miRNAs. RNA duplexes excised from pre-miRNAs are exported from the nucleus into cytoplasm (Rogers and Chen, 2013). Subsequently, one of the small RNA strands referred to as miRNA is stably incorporated into AGO1, the effector nuclease of the RNA-induced silencing complexes (RISCs). The other strand, known as miRNA^*^ is rapidly degraded. MiRNAs can regulate gene expression by guiding AGO1 to cleave target mRNAs with complementary target sites or to interfere with protein translation (Jones-Rhoades et al., 2006; Voinnet, 2009; Kamthan et al., 2015).

There are a number of publications describing the expression profiles of individual miRNAs in response to nutrient deficiency, only a few attempts have been made to comprehensively cover the molecular mechanisms where miRNAs are important for the adaptive responses. This review shed light on (i) recent progress in understanding the mechanism of N and P acquisition, assimilation and mobilization in plant; (ii) elaborate on how plants respond to N and P deficiency and in what ways miRNAs contribute to this physiological adaptation; and (iii) discuss the involvement of plant miRNAs in the crosstalk between N and P under limiting conditions.

## Mechanisms for N and P uptake and translocation

N is an essential element for plant development as it is a key component of other cellular constituents such as nucleic acids, proteins, chlorophyll, and phytohormones (Hawkesford et al., 2012). Plants take up N mainly in the forms of nitrate (NO^−^_3_), ammonium (NH^+^_4_) or urea from the soil, however, NO^−^_3_ is preferred form for most arable plants (Crawford and Forde, 2002). Synergistic association of legumes, actinorhizal plants and several C4 grasses with symbiotic microorganisms can produce NH^+^_4_ that fix atmospheric N_2_ via bacterial enzyme nitrogenase (Andrews et al., 2013). Two main phases of N uptake and usage during life cycle of plants have been well described in the literature. The first phase is during the vegetative stage where N is taken up, stored and assimilated into amino acids or other nitrogenous compounds. The second phase is the remobilization of assimilated N at senescence, where these compounds will be released and remobilized to reproductive organs to support the developing seeds (Kant et al., 2011a).

The N uptake process from the soil can be conducted either directly by roots or indirectly via mycorrhizal fungi (Andrews et al., 2013). Two types of N uptake systems are well defined in plants i.e., high affinity transport system (HATS) and low affinity transport system (LATS), which functions at low external N concentrations (1 μm–1 mM) and high external N concentrations (>1 mM), respectively (Kraiser et al., 2011). Studies on the molecular mechanism of N uptake and translocation revealed the involvement of a number of genes for these processes (Masclaux-Daubresse et al., 2010; Kant et al., 2011a; Xu et al., 2012; Krapp et al., 2014). Four members of nitrate transporter families involved in the NO^−^_3_ uptake process are described in Arabidopsis i.e., nitrate transporter 1/peptide (NPD), nitrate transporter 2 (NRT2), the chloride channel (CLC), and slow anion channel associated homologs (SLAC/SLAH) (Krapp et al., 2014). NPD is a recent nomenclature of nitrate transporter 1 (NRT1) family recently proposed by Léran et al. (2014) since NRT1 transporters has been reported to transport NO^−^_3_ and other substrates such as auxin, ABA, and glucosinolates. However, here the original gene names as these were initially named are referred. While NO^−^_3_ entering plant cells by facilitation of nitrate transporters, NH^+^_4_ from external source is taken up by ammonium transporters (AMT) (Crawford and Forde, 2002). Upon entering plant cells, NO^−^_3_ is converted to nitrite then to NH^+^_4_ and finally to amino acids through the action of nitrate reductase, nitrite reductase, glutamine synthetase, and glutamate synthase (Crawford and Forde, 2002). During reproductive growth, there are three pathways to release the nitrogenous compounds: the chloroplast degradation pathway; the vacuolar and autophagic pathway; and the ubiquintin-26S proteasome pathway (Liu et al., 2008). After degradation, the released amino acids will be loaded into phloem and remobilized to the developing seeds by facilitation of amino acid transporters completing the plant N cycle (Kant et al., 2011a).

Similar to N, P is also an essential macro-nutrient for plant growth and development. P plays a vital role as a key constituent of nucleic acids, phospholipids and the high energetic phosphate compounds ATP, ADP, or AMP (Marschner, 2012). Despite its central role in cellular processes, P availability in soil for plant uptake is very limited compared to other mineral elements (Ramaekers et al., 2010; Shen et al., 2011). Plants take up phosphate (Pi) in the form of PO^−^_4_ and PO^−2^_4_. Unfortunately, a majority of P existing in soil is insoluble due to its adsorption, precipitation with other cations or conversion into organic forms by microbes. Therefore, to maintain P homeostasis, plants have evolved a number of adaptive responses including activation of Pi transporters, modifications of root architecture, secretion of phosphatases and organic acids and symbiosis with mycorrhizal fungi (Raghothama, 1999; Poirier and Bucher, 2002; López-Arredondo et al., 2014).

The initial transport of Pi from soil into roots requires a mechanism allowing Pi movement against an extremely high endogenous Pi concentration gradient in root cells, which is usually 1000–10,000 times higher than external Pi concentration in the soil (Bieleski, 1973). Plants have several Pi transporters for acquisition of Pi when external Pi availability is low (Rausch and Bucher, 2002; López-Arredondo et al., 2014). Pi transporter genes have been identified and cloned in many crop species and each of them plays a specific role to maintain P homeostasis (Lin et al., 2009). Four Pi transporter families have been identified in Arabidopsis; PHT1, PHT2, PHT3, and PHT4 (Poirier and Bucher, 2002; Guo et al., 2008). Pi is taken up from soil to root cells via mediation of members of the high affinity PHT1 family, which employs an H^+^-gradient at the plasma membrane to modulate H^+^/Pi symport activity (Shen et al., 2011). PHT2 family transporters are located in chroloplast and affects whole plant Pi allocation (Versaw and Harrison, 2002). PHT3 family members are located in mitochondrial inner membrane (Poirier and Bucher, 2002) and PHT4 family transporters are located in chloroplasts, non-photosynthetic plastids, and the Golgi apparatus (Guo et al., 2008). In addition, genetic screens also identified several key genes involved in Pi acquisition and translocation in Arabidopsis. Among these are *PHOSPHATE1* (*PHO1*) involved in Pi loading in xylem vessels in roots (Hamburger et al., 2002) and *PHOSPHATE2* (*PHO2*) a negative regulator of Pi uptake (Aung et al., 2006; Bari et al., 2006). These Pi transporters, PHO1 and PHO2 work in coordination for the acquisition and translocation of Pi in plants.

## Plant miRNAs involvement under N deficient responses

Under N limiting conditions, miRNAs can be up- or down-regulated. Expression profiles of different miRNA families have been observed in various crop species such as maize (Xu et al., 2011; Trevisan et al., 2012; Zhao et al., 2013), rice (Cai et al., 2012; Yan et al., 2014), soybean (Wang et al., 2013), and Arabidopsis (Pant et al., 2009; Liang et al., 2012). Alteration in the expression pattern of these miRNAs results in plant adaptive responses to N limitation in the soil via mediation of the expression of their target genes (Zeng et al., 2014). Involvement of different miRNAs under a N limitation response is summarized in Table 1. Changes of the expression patterns of miRNAs have been shown to play crucial roles in modulating adaptive responses. These adaptations include enhanced N uptake and transport, changes in plant architecture, production of metabolites and radical scavengers, reduced growth and early flowering and modulation of metabolism (Fischer et al., 2013; Zeng et al., 2014; Sinha et al., 2015), as discussed in the following sub-sections.

**Table 1:** **Different miRNAs involved in N and P limitation with their target genes, functions, and tissue specific effects**.

**MiRNA family**	**Target gene or protein**	**Description of function**	**Involvement under low N**		**Involvement under low Pi**	
			**Plant tissue^a^**	**Plant species (reference^b^)**	**Plant tissue**	**Plant species (reference)**
156	SQUAMOSA PROMOTER BINDING PROTEIN-LIKE (SPL) transcription factors	Shoot development Delayed vegetative phase change	R (+)	Maize (1)	R (+)	Arabidopsis (2)
			R (+)	Arabidopsis (3)	R (+), L (−)	White Lupin (4)
157	SQUAMOSA PROMOTER BINDING PROTEIN-LIKE (SPL) transcription factors				N (+)	Common bean (5)
159	MYB, TCP transcription factors	Plant development	R (+)	Maize (1)	R (+), SM (−), L (−)	White Lupin (4)
					R (+)	Soybean (6)
160	Auxin response factors	Reduce auxin responsive activities and the vegetative growth	R (+)	Maize (7)	R (+), L (−)	White Lupin (4)
		Lateral and adventitious root development, signal transduction	R (+)	Arabidopsis (3)		
162	Dicer like proteins	Flower development	R (+)	Maize (1)		
164	NAC transcription factors	Accelerate senescence, N remobilization	L (+), R (−), S (−)	Maize (1, 7)	R (+), SM (−), L (−)	White Lupin (4)
166	HD-ZIP transcription factors	Shoot development	R (−)	Maize (8)	R (+), SM (−), L (−)	White Lupin (4)
167	Auxin response factors	Enhance auxin responsive activity; lateral root outgrowth Reduced fertility, impaired reproductive organ development	R (−)	Maize (7)	R (+), L (−)	White Lupin (4)
			R (+), S (+)	Maize (1)		
			R (−)	Arabidopsis (3)		
168	ARGONAUTE1	Homeostasis and feedback regulation on miRNAs	R (−)	Maize (7)	R (+), L (+)	White Lupin (4)
169	HAP2 transcription factorsCAAT binding factor/NFYA	Nitrogen homeostasis, stress response Nitrogen homeostasis, N uptake Antioxidant	R (−), S (−), L (−)	Maize (1, 7–9)	SD (−), R (−), S (−)	Arabidopsis (2, 10, 11)
			R (−), SD (−)	Arabidopsis (3, 10)	R (−), S (−)	Arabidopsis (2)
			R (−), S (−)	Arabidopsis (12)		
			R (−), S (−)	Soybean (13)		
171	SCARECROW-like transcription factors	Root development	R (+)	Arabidopsis (3)	SM (+), L (+)	White Lupin (4)
			R (+), S (+)	Maize (1)		
			R (−), S (−)	Soybean (13)		
172	AP2 like transcription factors	Ethylene-responsive pathway, N remobilization Flower development	L (+), S (+)	Maize (1, 7)	L (−)	Tomato (14)
			R (−)	Arabidopsis (3)		
319	TCP transcription factors	Reduce vegetative growth	R (−)	Maize (7)	R (+), SM (−)	White Lupin (4)
			R (−), S (−)	Soybean (13)	R (+), L (−)	Tomato (14)
					R (−)	Soybean (6)
393	Auxin receptors	Root development, defense response	R (+)	Maize (1)		
394	F-box protein	Shoot development	S (+)	Maize (1)		
			R (−), S (−)	Soybean (13)		
395	ATP sulfurylase; sulfate transporters	Sulfate homeostasis	R (−)	Arabidopsis (3)	R (−), S (−)	Arabidopsis (2)
			R (−)	Maize (1, 7)	R (−), SM (+), L (+)	White Lupin (4)
396	Growth Regulating Factor (GRF)	Leaf development	R (−)	Maize (1)	R (+), L (−)	White Lupin (4)
			R (+/−), S (+/−)	Soybean (13)		
397	Laccases	Reduce root growth Copper homeostasis	L (−), S (−), R (−)	Maize (1, 7)	L (−)	White Lupin (4)
			R (−)	Arabidopsis (3)	L (−)	Common bean (5)
			R (−); S (−)	Soybean (13)		
398	COX5b-1; CCS1COX	Copper homeostasis, oxidative stress Enhanced to produce ATP under stress	R (−), SD (−)	Arabidopsis (3, 10)	SD (−), R (−), S (−)	Arabidopsis (2, 10)
			L (−); S (−)	Maize (1, 7)	L (+)	Tomato (14)
			R (−), S (−)	Soybean (13)	R (−)	Soybean (6)
					L (−)	Common bean (5)
399	Ubiquitin conjugase E2/UBC24	Phosphate homeostasis, uptake and translocation	L (−), R (−)	Maize (1, 7)	SD (+), R (+), S (+)	Arabidopsis (2, 10, 11, 15, 16)
			R (−)	Arabidopsis (3)	R (+), L (+)	*Medicago truncatula* (17)
					R (+), L (+)	Common bean (18, 19)
					L (+)	White Lupin (4)
					R (+), L (+)	Tomato (14, 16)
					R (+), S (+)	Rice (15, 16, 20)
					S (+)	Barley (21)
408	PLANTACYANINLaccases	Enhance electron carrier activity Copper homeostasis	L (−), R (−)	Maize (1, 7, 8)		
			R (−)	Arabidopsis (3)		
			R (−); S (−)	Soybean (13)		
444	MADS-box	Root development	R (+)	Rice (24)	R (+)	Rice (24)
528	POD, SOD	Enhance to scavenge free radical and active oxygen species under -N	L (−), R (−), S (−)	Maize (1, 7, 8)		
778	SET domain containing protein				SD (+), R (+), S (+)	Arabidopsis (2, 10)
780	Na^+^/H^+^ antiporter	Sodium ion export	R (+)	Arabidopsis (3)		
826	Alkenyl hydroxalkyl producing 2	Glucosinolate synthesis	R (+)	Arabidopsis (3)		
827	Ubiquitin E3 ligase with RING and SPX	Nitrogen/phosphorus metabolism Accelerate leaf senescence, P homeostasis, P uptake	R (−)	Arabidopsis (3)	SD (+), R (+), S (+)	Arabidopsis (2, 10, 11, 22, 23)
			L (−), R (−)	Maize (1, 7)	R (+), S (+)	Rice (22)
					S (+)	Barley (21)
828	TAS4	Anthocyanin biosynthesis			S (+)	Arabidopsis (2)
857	Laccases	Copper homeostasis	R (−)	Arabidopsis (3)		
2111	F box protein				SD (+), R (+), S (+)	Arabidopsis (2, 10)

*^a^ Plant tissue: R, root; L, leaf; S, shoot; N, nodule; SM, stem; SD, seedling; (+), up; (−), down*.

*^b^ References are listed as follows: 1, (Zhao et al., 2012); 2, (Hsieh et al., 2009); 3, (Liang et al., 2012); 4, (Zhu et al., 2010); 5, (Valdés-López et al., 2010); 6, (Zeng et al., 2010); 7, (Xu et al., 2011); 8, (Trevisan et al., 2012); 9, (Zhao et al., 2013); 10, (Pant et al., 2009); 11, (Lundmark et al., 2010); 12, (Zhao et al., 2011); 13, (Wang et al., 2013); 14, (Gu et al., 2010); 15, (Bari et al., 2006); 16, (Chiou et al., 2006); 17, (Branscheid et al., 2010); 18, (Liu et al., 2010); 19, (Valdés-López et al., 2008); 20, (Zhou et al., 2008); 21, (Hackenberg et al., 2013); 22, (Lin et al., 2010); 23, (Kant et al., 2011b); 24, (Yan et al., 2014)*.

### Changes in root growth and development

Several miRNAs have been reported to play vital roles in root growth and development under N deficiency (Xu et al., 2011; Liang et al., 2012). N deficiency modifies root architecture and morphology to improve the plant's ability to acquire nutrients from the soil more efficiently (Hermans et al., 2006). Recent studies have demonstrated the regulatory roles of miRNAs on root architecture and growth under N deficient conditions (Khan et al., 2011; Liang et al., 2012; Zhao et al., 2012; Wang et al., 2013). In Arabidopsis, two Auxin Response Factors (ARF transcription factors), *ARF6* and *ARF8*, regulate development of reproductive organs and are targets of miR167 (Wu et al., 2006). Further it was shown that *ARF8* is the regulator of lateral roots, where its expression was induced in pericycle and lateral root cap cells under N limiting conditions (Gifford et al., 2008). ARF proteins bind to auxin responsive *cis*-acting promoter elements and can induce or suppress gene expression in response to the plant phytohormone auxin (Hagen and Guilfoyle, 2002; Liscum and Reed, 2002). MiR167 also reportedly targets *IAA-Ala Resistant3* (*IAR3*), whose protein hydrolyses the inactive auxin derivative indole-3-acetic acid alanine and releases bioactive auxin, for root development during high osmotic stress (Kinoshita et al., 2012). Over-expression of miR167 resulted in plant morphologies similar to *arf6* and *arf8* mutant phenotypes (Wu et al., 2006). Therefore, lower expression of miR167 under N deficiency might lift its inhibition on auxin transcription factors which could in turn induce lateral root growth (Liang et al., 2012). In contrary, miR160 was induced while *ARF16* and *ARF17* were down-regulated under N limitation (Liang et al., 2012). In Arabidopsis, *ARF16* modulates root cap cell formation while *ARF17* serves as a regulator of *GH3*-like early auxin response genes (Mallory et al., 2005; Wang et al., 2005). Studies showed that miR160 over-expressed transgenic plants had more developed lateral roots implying that induced expression of miR160 might promote lateral root growth via mediation of *ARF16* and *ARF17* under N deficiency (Liang et al., 2012). Further study demonstrated that modulation of the root system under N starvation was actually coordinated by a spatial regulatory complex of three miRNAs: miR160, miR167, and miR171 (Liang et al., 2012). Under N deficiency, root growth was increased by enhanced expression of miR160 and miR171 and reduced expression of miR167.

The *NAC* gene family encode transcription factors that play multiple roles in developmental processes in plants. *NAC*s consist of three gene families; *NAM* (No Apical Meristem), *ATAF* (Arabidopsis Transcription Activation Factor), *CUC* (CUp shaped Cotyledon) (Olsen et al., 2005). MiR164 was reported to target five NAC domain containing genes, including *NAC1* which is involved in auxin signal transduction for the growth and development of lateral roots (Guo et al., 2005) and *CUC1* which is required for normal embryonic, vegetative, and floral development (Mallory et al., 2004). Down-regulation of miR164 concomitant with the up-regulation of *NAC1* produced more lateral roots (Guo et al., 2005). A *NAC* locus has been reported to accelerate senescence and increase nutrient remobilization from leaves to the developing grains in wheat (Uauy et al., 2006). MiR164 is up-regulated in maize leaf under N limiting conditions (Xu et al., 2011). This might imply the role of miR164 in modulating both root and shoot development under N limitation adaptation.

### N uptake

MiRNA, miR169a is the only candidate reported so far, regulating the expression of key target N transporters under N limiting conditions. The MiR169 family in Arabidopsis consists of 14 members, among these miR169a is the main contributor to the total miR169 level (Zhao et al., 2011). In Arabidopsis, miR169 targets *NFY* (Nuclear Factor Y) a ubiquitous transcription factor consisting of 3 subunits A, B, and C, some of which bind to promoter regions and regulate expression of the nitrate transporters *AtNRT2.1* and *AtNRT1.1* (Zhao et al., 2011). Up-regulation of *NFYA5*, a target of miR169, reportedly enhanced drought tolerance by stimulating expression of several antioxidant genes (Li et al., 2008). Under N deficiency, miR169 was strongly down-regulated and its target *NFYA* family members were strongly induced in root and shoot tissues (Zhao et al., 2011). Furthermore, over-expression of *MIR169a* repressed expression of *NFYA* transcripts. These over-expresser transgenic plants were especially hypersensitive to N starvation, accumulating less N which resulted in leaf yellowing compared to the wild type (WT) plants (Zhao et al., 2011). The hypersensitivity of *MIR169a* over-expresser plants was associated with down-regulation of nitrate transporter genes *AtNRT2.1* and *AtNRT1.1* (*CHL1*) suggesting the regulatory role of miR169 in N uptake and remobilization. The *chl1* mutant plants showed a reduced expression of *AtNRT1.1* and its phenotype mimicked over-expressed *MIR169a* transgenic plants. These indicate that lower expression of miR169 is an adaptive response of plants under N limiting conditions.

### Production of secondary metabolites and radical scavengers

The role of miRNAs involved in the production of antioxidants and anthocyanins has been reported (Kandlbinder et al., 2004; Shin et al., 2005; Liang et al., 2012). These are secondary metabolites protecting plants from photo-inhibition damage under abiotic stresses including N limitation. In Arabidopsis, the *AOP2* gene which encodes 2-oxoglutarate-dependent dioxygenase and associates with glucosinolate biosynthesis is the target of miR826 (Liang et al., 2012). A recent study reported that under N limiting conditions miR826 was strongly induced while *AOP2* transcripts were significantly repressed (He et al., 2014). In addition, expression of the nitrate transporter (*NRT2.1*) and ammonium transporter (*AMT1.5*) genes were also induced (He et al., 2014). MiR826 over-expresser Arabidopsis transgenic plants showed enhanced tolerance under N limiting conditions, and had higher biomass, more primary and lateral roots, increased chlorophyll and less glucosinolate and anthocyanin contents (He et al., 2014). It can be hypothesized that these transgenic lines were able to withstand N limiting conditions better than WT plants and had less need to accumulate stress induced secondary metabolites such as glucosinolates and anthocyanin.

N deficiency was reported to repress expression of miR398 in plants (Pant et al., 2009; Liang et al., 2012). MiR398 is a conserved miRNA in Arabidopsis, rice, *Lotus*, and *Medicago* (Sunkar and Zhu, 2004). This miRNA targets transcripts of multiple genes: cytosolic *CSD1*, chloroplastic *CSD2, COX5b-1*, and *CCS1. CSD1* and *CSD2* encode a Cu/Zn superoxide dismutase (SOD) an important radical scavenger that protects plants from oxidative stress damage (Sunkar et al., 2006; Jagadeeswaran et al., 2009). *COX5b-1* encodes a subunit of the mitochondrial cytochrome *c* oxidase and *CCS1* encodes the copper chaperone for SOD (Beauclair et al., 2010; Zhu et al., 2011). Over-expression of *CSD2* was reported to confer tolerance to oxidative stress induced by high light (Sunkar et al., 2006). This suggests that down-regulation of miR398 might reduce its control on these target antioxidant genes and thus indirectly provided protection to the photosynthetic machinery from reactive oxygen species (ROS) generated from N deficiency (Kandlbinder et al., 2004; Shin et al., 2005).

In plants, miR156 targets transcripts of the Squamosa Promoter Binding Protein Like (SPL) family of transcription factors whose expressions were synergistically associated with anthocyanin biosynthesis (Gou et al., 2011). Over-expression of miR156 repressed the expression of *SLP*, concomitantly with an increased production of anthocyanin in Arabidopsis. The accumulation of anthocyanin and reduction of photosynthesis are adaptive responses of plants to N limiting condition, which protect them from photo-inhibition damage (Diaz et al., 2006; Peng et al., 2008). Thus, increased expression of miR156 might have resulted in higher levels of anthocyanin production conferring better protection of plants during N starvation (Liang et al., 2012).

### Modifications of flowering time

MicroRNAs have been long known for controlling flowering time in plants (Yamaguchi and Abe, 2012; Spanudakis and Jackson, 2014). MiR156 was shown to regulate flowering, vegetative phase changes, fertility, and leaf formation via mediation of the *SPL* genes (Wu and Poethig, 2006; Wang et al., 2008, 2009; Wu et al., 2009; Xing et al., 2010). Transgenic plants over-expressing miR156 resulted in a prolonged juvenile phase, stunted growth, altered biomass production, and delayed flowering (Wu and Poethig, 2006; Xie et al., 2006; Chuck et al., 2007a; Zhang et al., 2011b; Fu et al., 2012; Shikata et al., 2012). MiR172 targets the *AP2-like* family of transcription factors including *TOE1* and *TOE2* and controls flowering time and floral organ identity in maize and Arabidopsis (Aukerman and Sakai, 2003; Chen, 2004; Chuck et al., 2007b; Zhao et al., 2007). In contrast to miR156, miR172 over-expressers were shown to promote flowering in Arabidopsis (Aukerman and Sakai, 2003; Chen, 2004; Jung et al., 2011). Further research revealed that miR156 regulates expression of miR172 via mediation of the transcription factors *SPL9* and *SPL10* (Wu et al., 2009). Over-expressed *35S::miR156a* transgenic plants had only half of the normal transcript level of miR172, whereas *35S::MIM156* transgenic plants had more than double the miR172 level (Wu et al., 2009). Since N starvation is known to induce early flowering in plants (Vidal et al., 2014), the changes in the expression pattern of miR156 and miR172 under N deficiency (Liang et al., 2012) will likely result in modification of flowering time.

## Plant miRNAs involvement in Pi deficient responses

Differential expression patterns of miRNAs under Pi limitation have been observed in several plant species for example Arabidopsis (Hsieh et al., 2009; Lundmark et al., 2010), common bean (Valdés-López et al., 2010), soybean (Zeng et al., 2010), rice (Zhou et al., 2008; Lin et al., 2010; Yan et al., 2014), barley (Hackenberg et al., 2013), white Lupin (Zhu et al., 2010), tomato (Gu et al., 2010), and *Medicago truncatula* (Branscheid et al., 2010). Similar to their behavior in N limiting condition, changes in the level of miRNAs mediated the expression of target genes resulting in physiological and morphological changes of plants under low Pi conditions. These changes include modifications of root architecture, production of metabolites and biosynthesis of anthocyanin and oxidative radical scavengers. Involvement of different miRNAs under Pi deficiency is shown in Table 1.

### Root architecture changes

MiRNAs are known to regulate root architecture (Guo et al., 2005; Mallory et al., 2005). Their differential expression patterns under Pi limiting conditions (Zhu et al., 2010) suggest their regulatory role in modulating root growth. Pi starvation induces changes in plant root system architecture as adaptive responses such as minimizing development of primary roots, increasing root branching, and stimulating elongation of lateral roots (Péret et al., 2014). Expression of miR160, miR164, and miR167 were up-regulated specifically in roots and were down-regulated in stem and leaves under Pi starvation in white Lupin (Zhu et al., 2010). Studies on Pi deficient white lupin reported that plants produced more lateral roots (Johnson et al., 1996), which enable the plant to secrete more organic acids and thus to facilitate liberation of precipitated Pi from the soil (Massonneau et al., 2001). Since miR160, miR164, and miR167 modulate root growth via mediation of *NAC* and auxin transcription factors (Guo et al., 2005; Mallory et al., 2005) it is arguable that their induced expression locally in the roots might be associated with lateral root development in response to Pi deficiency.

### Pi uptake, relocation, and remobilization enhancement

A number of miRNAs, their target genes and involvement in Pi uptake, relocation, and remobilization has been well characterized. As discussed in the earlier section, reduced expression of miR169 facilitated N uptake, and increased expression of the nitrate transporter genes *AtNRT2.1* and *AtNRT1.1* via mediation of *NFYA* transcription factors. A recent study showed that increased supply of NO^−^_3_ also stimulated root formation thus enhancing Pi uptake in the shrub legume (Maistry et al., 2014). It seems likely that the down-regulation of miR169 under a low Pi condition (Hsieh et al., 2009; Pant et al., 2009) was an adaptive response to facilitate N uptake and remobilization, which in turn indirectly stimulates Pi uptake.

The role of miR399 in regulation of P homeostasis via Pi acquisition and translocation is well characterized (Chiou, 2007; Liu et al., 2014). MiR399 targets *PHO2* (or *UBC24*) gene which encodes a ubiquitin conjugating E2 enzyme (Aung et al., 2006; Bari et al., 2006). *PHO2* plays a role to negatively regulate the level of Pi uptake, translocation, and remobilization when Pi supply is sufficient. *pho2* mutant plants accumulate excessive amounts of Pi in shoots and thus exhibit Pi induced toxic symptoms in Arabidopsis (Delhaize and Randall, 1995; Aung et al., 2006). In agreement, miR399 over-expresser plants enhance Pi uptake and translocation in shoots and under Pi sufficient conditions causing Pi toxicity in plants (Fujii et al., 2005; Aung et al., 2006; Bari et al., 2006; Chiou et al., 2006). MiR399 was up-regulated and *PHO2* was down-regulated under low Pi conditions (Chiou et al., 2006) suggesting the role of miR399 in maintaining Pi homeostasis in plants. Recent findings showed that PHO2 is located in the endomembrane system and mediates the degradation of PHT1 family members and PHO1 (Liu et al., 2012; Huang et al., 2013). As remobilization of Pi from old to young leaves was inhibited under Pi starvation (Chiou et al., 2006), induced miR399 expression helped maintain Pi homeostasis via enhancing Pi uptake, transport, and remobilization from root to shoot by down-regulating its target gene *PHO2* (Kuo and Chiou, 2011). Other studies on plants such as common bean (Valdés-López et al., 2008; Liu et al., 2010) and rice (Hu et al., 2011) also reported similar interactions between miR399 and *PHO2* homologs in modulating Pi homeostasis, suggesting the regulatory roles of miR399 are conserved in angiosperms.

Similar to miR399, miR827 also plays a crucial role in maintaining Pi homeostasis in plants (Kant et al., 2011b; Lin et al., 2013; Liu et al., 2014; Park et al., 2014). MiR827 target the 5′-untranslated region of the *NITROGEN LIMITATION ADAPTATION* (*NLA*) transcripts (Kant et al., 2011b), which encode a RING type ubiquitin E3 ligase (Peng et al., 2007). The expression of miR827 is induced under a low Pi condition, where the transcript level of *NLA* was repressed (Kant et al., 2011b). Initially, *NLA* was reported to be involved in the adaptive response to N deficiency, where *nla* mutant plants showed earlier onset of senescence compared to WT plants under N limiting conditions and an inability to accumulate anthocyanins (Peng et al., 2007). However, later studies showed that a mutation in either *PHOSPHATE TRANSPORTER TRAFFIC FACILITATOR1* (*PHF1*) or *PHOSPHATE TRANSPORTER1:1* (*PHT1:1*) genes rescued the early senescence phenotype in *nla* mutant in response to N starvation. The early senescence phenotype of the *nla* mutant indeed was caused by excessive Pi accumulation similar to *pho2* mutant plants (Kant et al., 2011b). Pi over-accumulation was much more pronounced under low NO^−^_3_ conditions in both *nla* and *pho2* mutant plants suggesting the important role of *NLA* and *PHO2* in maintaining Pi homeostasis in a NO^−^_3_ dependent manner (Kant et al., 2011b). Further research showed that NLA is predominantly expressed in the plasma membrane. NLA; an E3 ubiquitin ligase interacts with PHO2; an E2 ubiquitin conjugase to degrade Pi transporter PHT1:4 by ubiquitination (Park et al., 2014). Up-regulation of miR399 and miR827 which are negative regulators of *PHO2* and *NLA*, respectively, would therefore enhance Pi uptake in plants under Pi limiting conditions (Kant et al., 2011b).

### Biosynthesis of anthocyanins and antioxidants

Pi deficiency induce accumulation of anthocyanins in plants and several miRNAs are known to target genes involved in the anthocyanin biosynthesis pathway. MiR828 is up-regulated under Pi deficiency and its target is *Trans-Acting SiRNA* gene 4 (*TAS4*) non-coding RNA transcript, which results in the production of tasi-RNAs (Hsieh et al., 2009). *TAS4-siR81* (−) one of the dominant *TAS4* siRNAs, targets a set of MYB transcription factors *PAP1/MYB75, PAP2/MYB90*, and *MYB113*, which regulate genes in the anthocyanin biosynthesis pathway and leaf senescence (Rajagopalan et al., 2006). MiR828 also target *MYB113*, suggesting an inter-relationship between these MYB genes, miR828 and *TAS4*. Since anthocyanin biosynthesis is also induced under Pi starvation (Misson et al., 2005), it is assumed that there is a mechanistic cross-talk between miR828, *TAS4-siR81* (−) and its target genes, which modulates anthocyanin accumulation. Pi starvation might activate expression of these MYB transcription factors leading to enhanced biosynthesis of anthocyanins. In addition, increased levels of *PAP1/MYB75* trigger the production of *TAS4-siR81* (−) via the activation of miR828 or *TAS4*, which in turn suppressed the expression of MYB transcription factors (Hsieh et al., 2009).

Oxidative stress conditions are a common phenomenon in plants under nutrient deficiency in general and under Pi limitation in particular (Kandlbinder et al., 2004; Shin et al., 2005). To cope with deleterious oxidative stress conditions, plants usually develop a protective mechanism by activating production of oxidant scavengers in order to maintain redox homeostasis. Under Pi starvation, two miRNAs, miR169 and miR398, were reportedly down-regulated (Hsieh et al., 2009; Pant et al., 2009) suggesting that their reduced expression might involve plant tolerance to oxidative stress conditions. Up-regulation of *NFYA5*, a target of miR169, reportedly enhanced drought tolerance where *NFYA5* over-expresser transgenic Arabidopsis had increased expression of several antioxidant enzymes such as glutathione *S*-transferase and peroxidases (Li et al., 2008). Most likely, down-regulation of miR169 allows for coping with the oxidative condition generated from Pi limiting conditions. In Arabidopsis miR398 targets two genes *CSD1* and *CSD2*, which encode Cu/Zn SOD antioxidant scavengers (Sunkar et al., 2006). Down-regulation of miR398 by oxidative stresses was reported to promote post-transcriptional mRNA accumulation of *CSD1* and *CSD2* and enhanced oxidative stress tolerance (Sunkar et al., 2006). Thus, down-regulation of miR398 could potentially increase expression of the antioxidant genes *CSD1* and *CSD2* enhancing plant tolerance to oxidative stress conditions caused by Pi starvation.

## Plant microRNAs involvement in crosstalk between deficient N and P

Amongst miRNAs identified so far, perhaps miR399 and miR827 are best characterized with their crucial roles in maintaining Pi homeostasis in plants (Liu et al., 2014). However, differential expression patterns of these miRNAs under N (Xu et al., 2011; Liang et al., 2012; Zhao et al., 2012) and P (Hsieh et al., 2009; Pant et al., 2009; Lundmark et al., 2010) limiting conditions suggest that their regulatory roles also depend upon the interaction between N and P. An adequate and balanced supply of nutrients is important to meet plant nutritional requirement, and efforts have been made to identify interactions amongst the response to different nutrients in general and between N and P in particular. It is clear that N and P uptake and assimilation in plants are not independent processes, but they interact with each other, in which the supply of one affects the other (Fageria, 2001). Increasing Pi supply was reported to increase nodulation and N fixation of subterranean clover by mediation of host plant growth (Robson et al., 1981). A recent study also shows that increasing N supply stimulates root formation thereby enhancing Pi acquisition in shrub legume (Maistry et al., 2014).

The form of N present can also have an effect on the type of interaction between N and Pi. For example, a competing interaction for uptake has been reported between NO^−^_3_ and Pi, both forms being anionic, while no antagonism for uptake was found between NH^+^_4_ and Pi (Kant et al., 2011b). Further, the suppression by high levels of NO^−^_3_ on Pi uptake was higher compared to the suppression by high levels of Pi on NO^−^_3_ (Kant et al., 2011b). The differential suppression by these ions could be due to their altered mobility in soil, since NO^−^_3_ diffusion is 3–4 times faster than that seen for Pi (Tinker and Nye, 2000). Higher NH^+^_4_ supply was reported to increase Pi uptake in some crop species (Riley and Barber, 1971; Gahoonia et al., 1992), while higher application of NO^−^_3_ was found to suppress Pi uptake (Kant et al., 2011b). The synergistic effects of NH^+^_4_ toward Pi uptake could be attributed to its ability to create changes in rhizosphere pH and alter root development, where supply of NH^+^_4_ reduces pH in rhizosphere therefore enabling the availability of externally soluble Pi; whereas supply of NO^−^_3_ increases pH in the surrounding areas of the roots affecting Pi uptake (Hinsinger, 2001; Jin et al., 2014). Rice plants supplemented with NH^+^_4_ were shown to have higher Pi content in both roots and shoots than those supplied with NO^−^_3_ (Zeng et al., 2012). Furthermore, Pi uptake in plants requires involvement of H^+^-ATPase proton pump and co-transporters (Raghothama, 1999; Poirier and Bucher, 2002). Involvement of root plasma membrane H^+^-ATPase in the adaptation of plants in response to Pi deficiency has been reported (Yan et al., 2002; Shen et al., 2006). Transgenic Arabidopsis plants overexpressing plasma membrane H^+^-ATPase absorbed more Pi under low Pi conditions compared to the WT plants (Shen et al., 2006). It was hypothesized that Pi deficiency contributed to the enhanced activity of plasma membrane H^+^-ATPase and H^+^ pump leading to acidification the rhizosphere (Zhang et al., 2011a) which in turn makes external Pi available for plants. Recent studies on effects of N on Pi acquisition reported that there is an association of involvement of plasma membrane H^+^-ATPase in stimulating Pi uptake by addition of NH^+^_4_ fertilizer.

Plants share common adaptation responses under N or Pi limiting conditions such as activation of high affinity transporters, development of lateral roots to facilitate uptake process, remobilization of nutrients from older leaves to young leaves and reproductive parts, retardation of growth and photosynthesis and production of antioxidant scavengers (Fang et al., 2009; Kant et al., 2011a). Under Pi limitation, NO^−^_3_ uptake and translocation from roots to shoots was reduced in different plant species such as tobacco (*Nicotinana tabacum* L.) (Rufty et al., 1990), barley (*Hordeum vulgare* L.) (Rufty et al., 1991), and soybean (*Glycine max* L.) (Rufty et al., 1993). Pi starvation also reduced the uptake of NH^+^_4_ (Taylor et al., 2010). It was proposed that the decrease in NO^−^_3_ uptake was resulted from the decrease of ATP pool (Rufty et al., 1993) and the feedback control mechanisms where uptake of a respective nutrient element not only depends on its presence but also on the availability of all other nutrients in rhizosphere (Amtmann and Blatt, 2009). Furthermore, Pi deficiency resulted in reduced activity of nitrate reductase in the roots of bean (*Phaseolus vulgaris* L.) (Gniazdowska and Rychter, 2000), an important enzyme of N assimilation pathway, which in turn inhibited N uptake from external source. Moreover, de Groot et al. (2003) suggested that reduced N uptake in tomato (*Lycopersicon esculentum* Mill.) under Pi limitation could probably be mediated by leaf cytokinin concentrations since low cytokinin levels might cause decrease in nitrate reductase activity. Transcriptional profiling of Arabiopsis and maize shed more light on this aspect. Low Pi supply was shown to reduce expression of nitrate reductase genes in Arabidopsis (Wu et al., 2003) and in maize (Schlüter et al., 2013). Therefore, it is reasonable to mention that genes in N and P metabolisms have orchestrated to maintain the nutrient balance under Pi limiting condition. As discussed in previous section miR399 and miR827 play important roles in maintaining Pi homeostasis in plants. Under Pi limitation, these miRNAs are up-regulated to release their inhibition on *PHO2* and *NLA*. As a result, induced expression of PHT1 transporters will increase the uptake and translocation of Pi from roots to shoots (Liu et al., 2014). In contrast, under low NO^−^_3_ supply Pi accumulation was higher in Arabidopsis plants (Kant et al., 2011b; Krapp et al., 2011) and maize leaves (Schlüter et al., 2012), which might be resulted from feedback control responses (Amtmann and Blatt, 2009). It is well documented that under Pi deprivation remobilization of soluble carbohydrates from leaves to roots increased to facilitate transporter activity and Pi uptake (Hermans et al., 2006; Karthikeyan et al., 2007). It is likely that increased root growth and high level of soluble carbohydrate accumulation in the roots under N limitation stimulate Pi accumulation in plants (Paul and Stitt, 1993). Furthermore, low NO^−^_3_ supply down-regulates many Pi starvation responsive genes (Schlüter et al., 2012, 2013). Probably down-regulation of Pi starvation responsive genes in these conditions is to alleviate unnecessary Pi uptake and accumulation in plants. Nevertheless, this mechanism requires further research. Under N limitation, expression of miR399 and miR827 are significantly down-regulated to stimulate expression of *PHO2* and *NLA*, which are negatively correlated with PHT1 activity and Pi uptake. Since low NO^−^_3_ supply causes Pi accumulation in different plants (Kant et al., 2011b; Krapp et al., 2011; Schlüter et al., 2012), down-regulation of miR399 and miR827 under such conditions is possibly a counter measure to control the over-accumulation of Pi to the toxic level.

**Figure 1:**
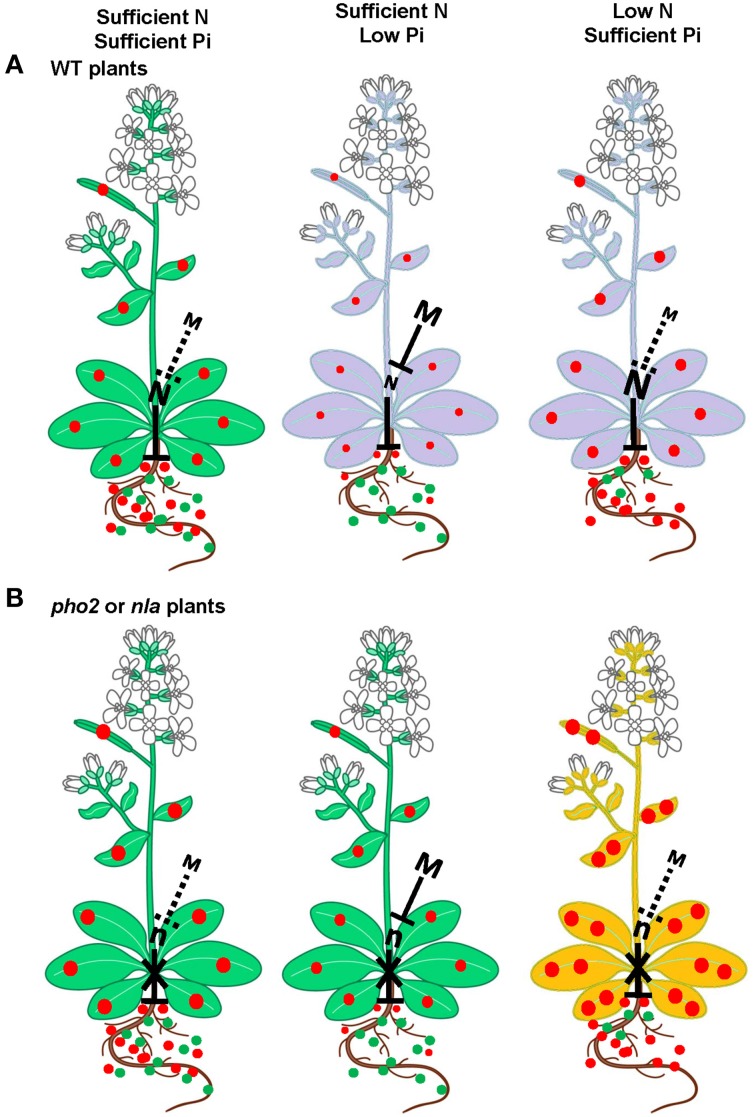
**Hypothetical model for the role of miR399 and miR827 to maintain Pi homeostasis in plants**. **(A)** In WT plants, *PHO2* and *NLA* act as repressors for Pi uptake and these genes are targets of miR399 and miR827, respectively. Under sufficient N and Pi supply plants grow normal and under low N or Pi supply plants accumulate anthocyanin. **(B)** In *pho2* or *nla* mutants, the *PHO2* or *NLA* gene are not functional and these plants have higher Pi uptake in general compared to WT plants. Under sufficient N and Pi supply, mutant plants grow normal like WT. Under sufficient N and low Pi supply, since *PHO2* or *NLA* gene are non-functional thereby letting more Pi uptake resulting in normal plant growth of these plants. Low N and sufficient Pi conditions accelerate excessive Pi accumulation causing Pi toxicity in these mutants. Purple leaves show N or Pi deficiency leading to anthocyanin accumulation, orange leaves indicate Pi toxicity. Dotted lines indicate less suppression. The size of the letter M or N correlates with expression level. The size and number of the solid green and red circles correlates with N and Pi concentration, respectively. M, miR399 or miR827; N, PHO2 or NLA (adapted from Kant et al., 2011b).

A hypothetical model for the crosstalk between N and P with emphasis on the role of miR399 and miR827 and their target genes *PHO2* and *NLA* is presented in Figure 1, which is based on the report of Kant et al. (2011b). They studied a range of combinations of N and P applications and has described under which combinations *pho2* and *nla* mutants were showing Pi toxicity. There was a strong effect of interaction between N and P supply on both *pho2* and *nla* phenotypes. When supply of N and Pi is sufficient, both WT and *pho2* and *nla* mutant plants show a normal phenotype (Figure 1A_Sufficient N and Pi). Pi toxicity effect was not observed in *pho2* and *nla* mutant plants although Pi concentration was two-fold higher in these plants. Probably Pi level might not reached the critical toxic point (Figure 1B_Sufficient N and Pi) (Delhaize and Randall, 1995; Kant et al., 2011b). Under low Pi supply, miR399 and miR827 expressions are induced with concomitant repression of *PHO2* and *NLA* genes though these genes are still functional in WT plants imposing some repression on Pi uptake. Lower Pi supply and uptake result in anthocyanin accumulation in WT plants, a known phenomenon (Figure 1A_Low Pi) (Jiang et al., 2007; Kant et al., 2011b). Interestingly, in *pho2* or *nla* mutant plants *PHO2* and *NLA* genes are non-functional thereby letting more Pi uptake compared to WT plants resulting in normal growth of these plants (Figure 1B_Low Pi). Under low N supply, WT plants accumulate anthocyanin; a known phenomenon (Figure 1A_Low N) (Peng et al., 2007, 2008; Kant et al., 2011b). Expressions of miR399 and miR827 was reduced with enhanced expression of *PHO2* and *NLA* genes regulating the controlled Pi uptake (Figure 1A_Low N). Since these genes are non-functional in *pho2* or *nla* mutant plants, Pi uptake is not regulated resulting in excessive accumulation of Pi causing Pi toxicity in *nla* or *pho2* mutant plants (Figure 1B_Low N) (Delhaize and Randall, 1995; Kant et al., 2011b).

## Conclusions and future perspectives

It is important for both economic and environmental reasons to improve N and P use efficiency in plants. Improving N and P uptake under their limiting supply would be a viable approach to utilize these nutrient elements more efficiently. The strategies for such improvement could include optimizing agricultural practices, molecular marker assisted breeding, and genetic engineering of genes involved in N and Pi uptake and metabolism (Ramaekers et al., 2010; McAllister et al., 2012; Guevara et al., 2014; López-Arredondo et al., 2014; Dass et al., 2015). A genetic engineering approach by manipulation of miRNA expression to improve N use efficiency was proposed (Fischer et al., 2013; Sinha et al., 2015), a similar approach is quite reasonable to propose for the improvement of P use efficiency.

Since the cloning of first plant miRNA in 2002 (Reinhart et al., 2002), a number of miRNAs have been identified. In the past five years, the number of newly cloned plant miRNAs has increased from 10,898 to 15,041 and target transcripts have been extended to 178,138 in 46 species (Yi et al., 2015). Several genetically engineered plants using different miRNAs have shown improved resistance against biotic and abiotic stresses (Kamthan et al., 2015). Still the understanding of the functionality of several miRNAs is unclear or their target genes have unknown function. Only a few of the miRNAs and their target genes have been completely characterized and experimentally validated especially those involved in adaptive response to N and Pi limitation conditions. Further research and studies are required and would be much helpful to decipher the regulatory roles of known miRNAs and their target gene functions involved in plant growth and development in general and improving efficient utilization of key macro-nutrient elements in particular.

To understand the role of miRNAs and involvement in crosstalk between N and P in plant is quite important. However, this interactive mechanism remains elusive and requires further investigations. The uptake and utilization processes of N and P in plants are complex and cannot be considered in isolation. Instead these processes are synergistic and depend on the nutritional requirements of the plants in a particular environment. Furthermore, levels of N and P cause affect not only the root and associated transport systems in plants but also the ion balance of other macro- (K, Ca, Mg, and S) and micro-nutrients (Mn, Fe, Zn, and Cu, etc.) (Schlüter et al., 2013).

Current knowledge of the involvement of various miRNAs for the regulation of N and P uptake, assimilation and utilization and plant adaptation to both N and P limitation conditions has been reviewed here along with how these processes affects the modifications in shoot and root growth, development, and architecture, effects on vegetative and reproductive phase and production of secondary metabolites. Nevertheless, a holistic approach to study the interaction of N and P along with other macro- and micro-nutrient elements and the involvement of miRNAs would be of much benefit and would thus require further research.

### Conflict of interest statement

The authors declare that the research was conducted in the absence of any commercial or financial relationships that could be construed as a potential conflict of interest.
